# Effects of Steel Slag, Desulfurization Gypsum, and Ground Granulated Blast-Furnace Slag on the Characterization of Recycled Cement-Stabilized Macadam

**DOI:** 10.3390/ma18040874

**Published:** 2025-02-17

**Authors:** Haoyu Tan, Henggang Ji, Peilong Yuan, Xiang Fan

**Affiliations:** 1 CCCC-SHEC Dongmeng Engineering Co., Ltd., Xi’an 710076, China; 2 School of Highway, Chang’an University, Xi’an 710064, China; jihenggang@chd.edu.cn (H.J.); fanxiang224@126.com (X.F.)

**Keywords:** industrial solid waste, recycled concrete aggregate, microscopic characteristics, synergy of mixed cementitious materials, reuse of solid waste

## Abstract

Steel slag powder (SS), ground granulated blast-furnace slag (GGBS), and flue gas desulfurization gypsum (FDG) are environmentally friendly and cost-effective substitute materials for ordinary Portland cement (OPC). This study investigated the use of industrial solid wastes, including SS, GGBS, and FDG, as auxiliary materials in OPC to stabilize pretreated recycled concrete aggregate (pretreated RCA). The use of pretreated RCA, mixed cementitious materials, and water at the optimum content created a mixture designated recycled cement-stabilized macadam (RCSM). A series of mechanical tests were conducted to clarify the performance of the RCSM, and microscopic tests were performed to elucidate the microcharacteristics of the mixed cementitious materials. With a curing time from 3 days to 28 days, the unconfined compression strength (UCS) of the mixed cementitious materials (A4) composed of SS, GGBS, FDG, and OPC increased by 5.94–10.79% compared with that of the cementitious material of OPC (A0). The UCS of the mixture composed (C4) of SS, GGBS, FDG, OPC, and pretreated RCA was greater than that of the mixture composed (C0) of OPC and RCA from 7 days to 90 days, increasing by 4.26–8.35%. The total drying shrinkage coefficient of C4 was lower than that of C0, whereas the temperature shrinkage coefficient of C4 was higher than that of C0, indicating that the use of A4 can effectively reduce drying shrinkage cracking in C4. The hydration products of A4 primarily consisted of flocculent calcium silicate hydrate (C-S-H) gel, fibrous calcium aluminate hydrate gel, and needle-like ettringite crystals. The interlocked growth of C-S-H gel and ettringite crystals continued and promoted an increase in the UCS of the cementitious system. The test results provide a reference for the application of similar materials.

## 1. Introduction

With the rapid development of related industries such as metallurgy and mining, the discharge of industrial solid waste is increasing. The types and quantities of industrial solid wastes are numerous, and the task of resource utilization is arduous [1,2]. Countries are eager to improve the utilization of industrial solid waste to achieve a good and healthy ecological environment. The cement industry is one of the main sources of carbon emissions, and reducing the use of cement can alleviate the greenhouse effect. Therefore, using industrial solid waste as an auxiliary cementing material to replace part of the cement is beneficial for improving the utilization of industrial solid waste and protecting the ecological environment.

Current research on the utilization of solid waste in cement-based materials indicates that alkaline solid waste has a very low utilization rate, primarily due to its high alkalinity, low content of silicon and aluminum compounds, and limited reactivity [3,4,5]. Similarly, sulfate solid waste has a low utilization rate due to its elevated sulfate content [6,7]. Additionally, silicon–aluminum solid waste has a relatively low utilization rate, which can be attributed to the low reactivity of its silicon and aluminum constituents [8,9,10]. The main mineral components of steel slag powder (SS) include dicalcium silicate (C_2_S), tricalcium silicate (C_3_S), and tetracalcium aluminoferrite, which are substances similar to the components of cement clinker. However, the reactivity of the SS is significantly lower than that of cement clinker [11]. The reactivity of SS can be enhanced to a certain degree through mechanical grinding, which increases its specific surface area [4]. To further stimulate the reactivity of SS, chemical activation was implemented following mechanical processing. Flue gas desulfurization gypsum (FDG) is a sulfate-based activator that promotes the depolymerization of the glass phase of SS, generating calcium silicate hydrate (C-S-H) gel and calcium aluminate hydrate (C-A-H) gel [6]. These gels, in conjunction with the SO_4_^2−^ component, facilitate the formation of ettringite crystals. Moreover, the addition of GGBS can enhance the hydration of SS. The hydration reaction of GGBS produces a significant amount of C-S-H gel, which necessitates the consumption of Ca^2+^. Consequently, in the GGBS and SS composite cementing system, the hydrolysis and hydration processes of both materials exhibit mutually beneficial effects [9].

Hao et al. [12] investigated the hydration products of FDG and SS composite cementitious materials, which exhibited a distinct C-S-H gel structure, which helped to enhance the strength of the material, thereby improving its mechanical properties. Zhang et al. [13] reported that industrial solid wastes, including SS, FDG, and desulfurized ash, were utilized as the primary raw materials to prepare binder materials. The combined action of OH^−^ and SO_4_^2−^ facilitated the depolymerization of the SiO_4_ and AlO_4_ tetrahedrons, which subsequently repolymerized. Due to the significant presence of the glass phase (SiO_4_ and AlO_4_ tetrahedrons) in GGBS, along with its high content of oxides such as SiO_2_, Al_2_O_3,_ and CaO, the method of alkali excitation can be employed to transform the SiO_2_ and Al_2_O_3_ in GGBS into highly reactive silicon–aluminum compounds exhibiting gel properties. Liu et al. [14] also reported that under the alkaline conditions provided by SS, SiO_4,_ and AlO_4,_ tetrahedrons continuously dissociated from GGBS, leading to the further development of C-S-H gel and ettringite crystals. Niu et al. [15] investigated an environment provided by OH^−^ from SS and SO_4_^2−^ from FDG and reported that GGBS was activated and underwent hydration, resulting in the formation of C-S-H gel and ettringite crystals. In addition, alkaline activators also have a positive effect on the performance of cement. The mechanical properties of alkali-activated recycled cement with a replacement rate of up to 30% were significantly improved compared to non-alkali-activated recycled cement [16,17].

Compared with other semirigid base materials, cement-stabilized macadam (CSM) offers several advantages, including moisture resistance and high strength [18,19,20]. CSM is a composite material composed of aggregates with proper gradation, 3–8% of the cementitious materials of the aggregate weight, and water at the optimum content [21]. Buildings constructed in the early stages face renovation or reconstruction, and the disposal of abandoned concrete has become a challenge. Therefore, the utilization of waste concrete as recycled concrete aggregate (RCA) not only solves the resource problem but also saves land for waste concrete disposal. Considering that the compressive strength requirement of CSM is generally lower than that of ordinary cement concrete and that the aggregate demand of CSM is large, the use of RCA can meet the construction requirements of recycled cement-stabilized macadam (RCSM) for semirigid pavements. However, although the use of RCSM is promising, the incorporation of RCA introduces certain disadvantages. In particular, RCA has characteristics such as low density, high porosity, a rough surface texture, elevated water absorption, and the presence of numerous microcracks [22,23]. Consequently, the application of RCSM must consider the mechanical properties of RCA as well as the methods of surface treatment employed.

Common surface treatment methods for RCA can be classified into two categories [24,25]: the first involves the mechanical and acid solution removal of cement mortar from the RCA surface, whereas the second employs cementitious material to densify the existing cement mortar on the RCA surface. Katz et al. [26] investigated the treatment of RCA by impregnating a silica fume solution and ultrasonic cleaning. Following the impregnation of the silica fume, increases in compressive strength of 30% at 7 days and 15% at 28 days were observed, whereas ultrasonic cleaning yielded an improvement of approximately 7%. Tam et al. [27] employed three types of presoaking treatment approaches to remove the mortar adhering to the surface of RCA. The resulting concrete demonstrates significant improvements in compressive strength, flexural strength, and the modulus of elasticity compared with those of concrete produced using traditional methods. Tam et al. [28] reported that treatment with silica fume can enhance the microstructure of RCA. Silica fume effectively fills the pores and voids within the adhered cement mortar and subsequently reacts with calcium hydroxide to form a C-S-H gel. The residual cement mortar on the surface of the RCA has a certain effect on the performance of the mixture. To better apply RCA in RCSM, it is necessary to treat the RCA in advance.

The RCA is immersed in a solution formulated from GGBS, SS, FDG, and cement to enhance its performance. The main purpose of this study is to explore the use of pretreated RCA in conjunction with mixed cementitious materials to create a mixture designated as an RCSM. To achieve these objectives, mixture performance tests, including crushing value tests, unconfined compressive tests, splitting tensile tests, compressive modulus tests, splitting modulus tests, drying shrinkage tests, and temperature shrinkage tests, were applied. To further clarify the interactions of mixed cementitious materials, X-ray diffraction tests, scanning electron microscopy tests, and mercury intrusion porosimetry tests were conducted. This study provides new solutions for the utilization of industrial solid waste and RCA in recycled cement-stabilized macadam.

## 2. Materials and Testing Methods

### 2.1. Cementitious Materials

#### 2.1.1. Materials

**Figure 1:**
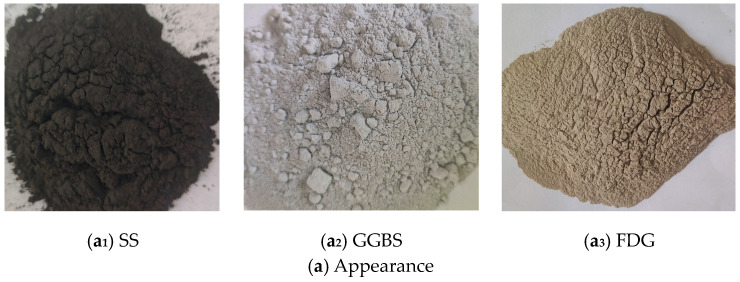

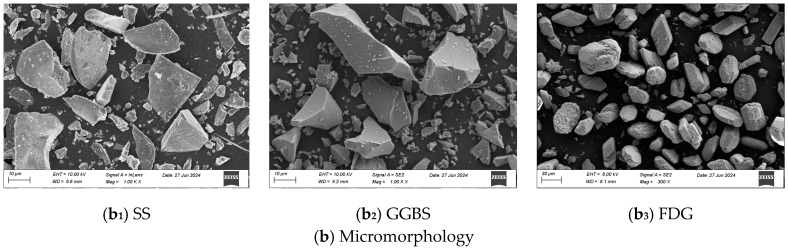
Appearance and micromorphology of SS, GGBS, and FDG.

**Figure 2:**
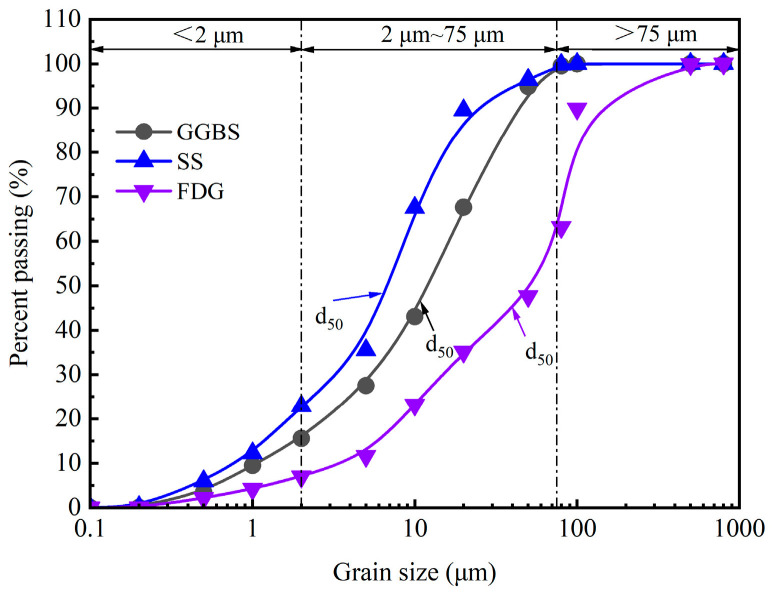
Particle size distribution curves of SS, GGBS, and FDG.

**Figure 3:**
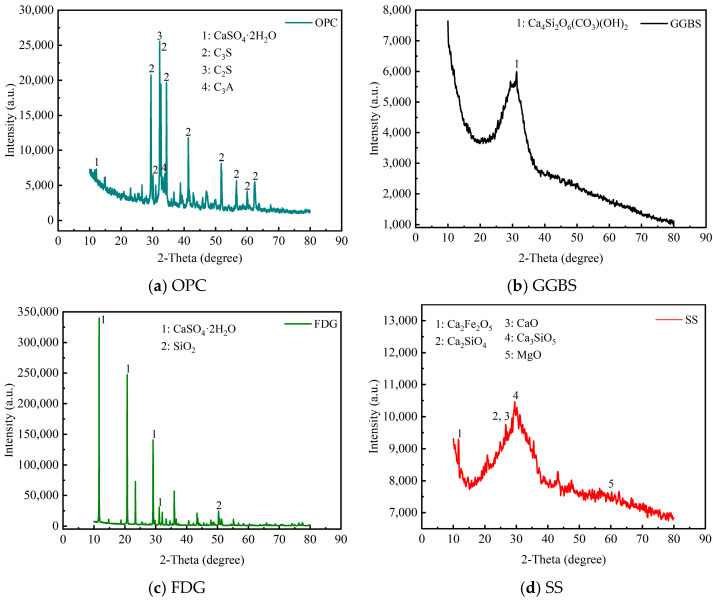
XRD analysis of OPC, SS, GGBS, and FDG.

The mixed cementitious material is composed of #42.5 ordinary Portland cement (OPC) and industrial solid waste, which includes steel slag powder (SS), ground granulated blast-furnace slag (GGBS), and gas desulfurization gypsum (FDG). The SS and OPC samples were sourced from Zhejiang Province, China, whereas the GGBS and FDG samples were obtained from Hebei Province, China. In this study, the OPC met the requirements set forth by the Chinese test standard GB 175-2023 [29], as illustrated in Table 1. The appearance, micromorphology, and physical properties of SS, GGBS, and FDG are presented in Figure 1 and Table 2, respectively. The SS exhibited a black color and an irregular shape, featuring micropores on its surface. In contrast, the GGBS was gray–white and irregular in shape but had a smooth surface. The FDG appeared light brown–yellow, was predominantly rectangular in shape, and possessed micropores on its surface. The particle size distributions of SS, GGBS, and FDG were measured with a Bettersize 2000LD laser particle size distribution meter, as shown in Figure 2. The laser particle size distribution meter used in this study is manufactured by Dandong Baite Instrument Co., Ltd., which is located in Dandong City, Liaoning Province, China. The primary chemical compositions (Table 3) and mineral compositions (Figure 3) of the materials were analyzed using X-ray fluorescence spectroscopy and X-ray diffraction (XRD). Notably, GGBS was predominantly amorphous, characterized by a single crystalline peak corresponding to the crystalline phase of Ca_4_Si_2_O_6_(CO_3_)(OH)_2_. The SS contained many crystalline phases, including C_2_S, C_3_S, CaO, MgO, and RO phases [30,31]. Chemical analysis revealed that SS and GGBS were primarily composed of silica, alumina, and calcium oxide, along with various other heavy metal oxides. The combined percentages of CaO, SiO_2_, and Al_2_O_3_ in SS and GGBS were 63.61% and 86.3%, respectively. Additionally, chemical analysis indicated that FDG was primarily composed of calcium oxide and sulfur trioxide, along with various other heavy metal oxides. The combined percentage of CaO and SO_3_ in the FDG was 93.8%. SiO_2_ and Al_2_O_3_ in SS and GGBS contribute to a hydration reaction when mixed with OPC [32].

#### 2.1.2. Design and Testing Methods for Mixed Cementitious Materials

Table 4 shows the design and test methods for mixed cementitious materials. The mixed cementitious materials consist of binder materials and water, with a mass ratio of water to binder materials of 0.45. A0 was the control group, and A1, A2, A3, and A4 were the test groups. The compressive strength of the mixed cementitious materials was tested in accordance with ASTM C 109/C 109 M-2001 [33]. The tests were conducted using a YAW-300D cement compressive–flexural testing machine with a continuous loading rate of 0.5–1.0 kN/s. The cement compressive-flexural testing machine used in this study is manufactured by Zhejiang Yingsong Instrument Equipment Manufacturing Co., Ltd., which is located in Shaoxing City, Zhejiang Province, China. A suspension solution was prepared with a mass ratio of water to binder materials of 5:1 for pH testing [34,35]. The pH of the suspension was measured using a pH-3C device at intervals of 2, 4, 6, 8, 12, 18, 24, 30, 42, 54, 66, 78, and 90 h.

### 2.2. Surface Treatments of Recycled Concrete Aggregate

Recycled concrete aggregate was sourced from Zhejiang Province, China. These aggregates were obtained through the crushing of waste concrete. The particle sizes of the coarse aggregates were classified into three ranges: 4.75–9.5 mm, 9.5–19 mm, and 19–26.5 mm. The fine aggregates were sourced from manufactured sand within the size range of 0–4.75 mm. The physical properties of the coarse aggregates were measured in accordance with the Chinese test standard JTG 3432-2024 [36], as shown in Table 5. The aggregate gradation was chosen based on the Chinese standard JTG/T F20-2015 [37]. The gradation of the aggregate is shown in Table 6.

**Figure 4:**
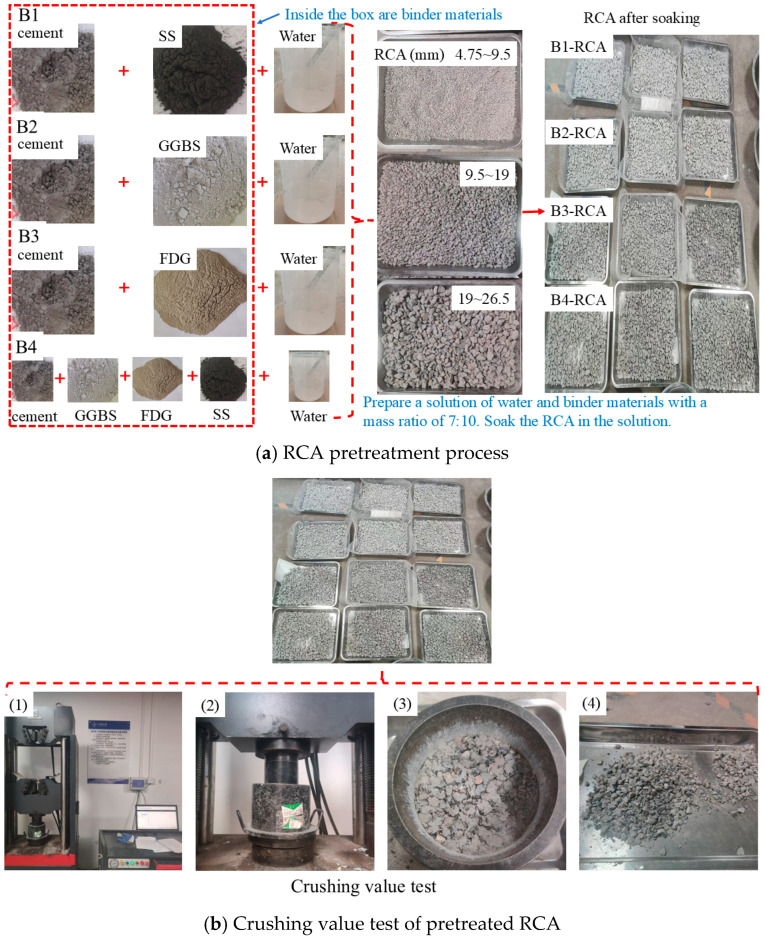
RCA pretreatment process and crushing value test.

Table 7 shows the pretreatment scheme for RCA. A solution with a 7:10 mass ratio of water to binder materials was prepared, and the RCA was soaked in the solution. The RCA (sized between 4.75 and 26.5 mm) was mixed with the prepared solution for 3 min to ensure complete coverage of the aggregate, followed by a soaking period of one hour. After soaking, the RCA was removed from the solution and cured under natural conditions for 3 days and 28 days. The recycled coarse aggregate, after curing for 28 days, was selected to prepare the mixed material samples. Figure 4 illustrates the pretreatment process of the RCA as well as the crushing value test.

Table 8 shows the crushing value results of the RCA following pretreatment. The range of aggregate particle sizes selected for the crushing value tests was 9.5–13.2 mm. Compared with that of the untreated aggregates, the crushing value of the RCA decreased by 10.9–22.4% after curing for 3 days and by 23.5–37.6% after curing for 28 days.

### 2.3. Performance Test of Recycled Cement-Stabilized Macadam

#### 2.3.1. Design of the Mixture

Test group: Mixed cementitious materials and pretreated RCA were used as RCSM. In the control group, OPC and RCA were used as RCSM. We selected a 5% dosage of mixed cementitious materials (A0, A1, A2, A3, and A4) based on our engineering experience. The aggregates used in this study include untreated RCA (B0-RCA) and pretreated RCA (B1-RCA, B2-RCA, B3-RCA, and B4-RCA).

Throughout the experiments, test method T0804-1994 was followed for the solidification of inorganic binder-stabilized materials in the (JTG 3441-2024) [38]. The optimum moisture content and maximum dry density of the mixtures (C0, C1, C2, C3, and C4) were determined using the center of gravity compaction method. C0 was the control group, and C1, C2, C3, and C4 were the test groups.

Table 9 presents the optimum moisture content and maximum dry density of the mixture. As shown in Table 9, the OPC content in mixtures C1, C2, C3, and C4 constituted 70% of the total mixed cementitious material. The variations in the maximum dry density and optimum moisture content of C1, C2, C3, and C4 were influenced by the proportions of SS, GGBS, and FDG within the mixed cementitious material. This situation might have been related to the specific surface area, normal consistency, and specific gravity. As indicated in Table 2, the specific surface area and normal consistency were ranked from largest to smallest as follows: SS > GGBS > FDG, whereas the apparent specific gravity was ranked in the opposite order. Table 8 indicates that the water absorption of the pretreated RCA was ranked from largest to smallest as follows: B1-RCA > B3-RCA > B2-RCA > B4-RCA. Additionally, among mixtures C1, C2, C3, and C4, C1 presented a higher water requirement, a greater optimum moisture content, and a lower maximum dry density.

#### 2.3.2. Sample Preparation

The tested items, sample sizes, and curing ages are presented in Table 10. The samples were formed using a WAW-1000B universal material testing machine under conditions of maximum dry density, optimal moisture content, and a specified compaction degree of 98%. The universal material testing machine and the cement compressive-flexural testing machine both come from the same company. Cylindrical samples with a diameter of 150 mm and a height of 150 mm were prepared for tests on unconfined compressive strength, the compressive modulus of resilience, splitting tensile strength, the splitting modulus of resilience, and freeze–thaw cycles. Additionally, beam samples measuring 100 mm in width, 100 mm in height, and 400 mm in length were prepared for drying shrinkage and temperature shrinkage tests. Mold release occurred after one day of sample curing. Following demolding, all the cylindrical and beam samples were immediately sealed in plastic bags to minimize moisture loss. The samples were subsequently placed in a standard curing room maintained at a relative humidity of 96% and a temperature of 20 ± 2 °C to reach the curing time.

#### 2.3.3. Test Method for Unconfined Compressive Strength

The test method for unconfined compressive strength was based on the T0805-2024 standard of JTG 3441-2024 [38]. The strain-controlled loading rate was set at 1 mm/min. The equation used to calculate the unconfined compressive strength (*R_c_*) is provided below:

(1) $$R_{c}=P/A=4P/\pi D^{2}$$

where *R_c_* is the unconfined compressive strength (MPa); *A* is the cross-sectional area of the sample (mm^2^); *P* is the maximum pressure when the sample is damaged (N); and *D* is the diameter of the sample (mm). Each group consisted of six sample replicates, and the final unconfined compressive strength was determined from the average strength of these six samples.

#### 2.3.4. Test Method for Determining the Splitting Tensile Strength

The test method for determining the splitting tensile strength was based on the T0808-1994 standard in JTG 3441-2024 [38]. The strain-controlled loading rate was set at 1 mm/min. The equation used to calculate the indirect tensile strength (*R_i_*) is provided below:

(2) $$R_{i}=2P/\pi Dh$$

where *R_i_* is the splitting tensile strength (MPa); *h* is the height of the sample (mm); *P* is the maximum pressure when the sample is damaged (N); and *D* is the diameter of the sample (mm). Each group consisted of six sample replicates, and the final splitting tensile strength was determined from the average strength of these six samples.

#### 2.3.5. Test Method for Determining the Compressive Modulus of Resilience

The compressive modulus of resilience test method was conducted according to the T0808-1994 standard, which was derived from JTG 3441-2024 [38]. After curing for 28 and 90 days, the samples were tested using the top surface method. The strain-controlled loading rate was set at 1 mm/min. The preset compressive stress was divided into five segments, and five loading and unloading tests were performed. The loading pressures applied were 0.2, 0.4, 0.6, 0.8, and 1.0 MPa. The equation used to calculate the compressive modulus of resilience (*E_c_*) is provided below:

(3) $$E_{c}=ph/l$$

where *E_c_* is the compressive modulus of resilience (MPa); *p* is the calculated unit pressure (MPa); *l* is the resilience deformation of the sample (mm); and *h* is the height of the sample (mm). Each group consisted of six sample replicates, and the final compressive modulus of resilience was determined from the average strength of these six samples.

#### 2.3.6. Test Method for the Splitting Modulus of Resilience

The test method for the splitting modulus of resilience was measured according to the T0852-2009 method of JTG 3441-2024 [38]. The samples were tested for the splitting modulus of resilience after curing for 28 and 90 days. The strain-controlled loading rate was set at 1 mm/min. The preset compressive stress was divided into five segments, and five loading and unloading tests were performed. The loading pressures applied were 0.1 MPa, 0.2 MPa, 0.3 MPa, 0.4 MPa, and 0.5 MPa. The equation used to calculate the splitting modulus of resilience (*E_s_*) is provided below:

(4) $$E_{s}=\frac{\left(p-p_{0})\times (0.27+1.0\mu )\times (1.794-0.0314\mu \right)}{D\times l_{y}\times (0.135+0.05\mu )}$$

where *E_s_* is the splitting modulus of resilience (MPa); *p* represents the different load levels (N); *p*_0_ is the initial load (N); *μ* is the Poisson’s ratio, which is 0.25; *D* is the diameter of the sample (mm); and *l_y_* is the difference between the average reading when loading and the average reading after unloading.

#### 2.3.7. Test Method of the Freeze–Thaw Cycle

The slow freezing method (T0858-2009) mentioned in the JTG 3441-2024 standard was used to evaluate the frost resistance of the laboratory samples [38]. The samples were subjected to freeze–thaw cycles after 90 days. For the freeze–thaw cycle, a set of samples was placed in a cryostat at a constant temperature of −18 °C for 16 h. Immediately following the freeze test, the samples were thawed in a temperature-controlled flume maintained at 20 °C for 8 h. The freeze resistance index of the samples can be calculated using the following formula:

(5) $$BDR=R_{DC}/R_{C}$$

where *BDR* is the residual strength ratio (%); *R_DC_* is the unconfined compressive strength of the sample after five freeze–thaw cycles (MPa); and *R_c_* is the unconfined compressive strength of the control sample without freeze–thaw cycles (MPa).

#### 2.3.8. Test Method for Drying Shrinkage

The drying shrinkage test method was executed with reference to the T0854-2024 method of JTG 3441-2024 [38]. The drying shrinkage test was conducted in a drying oven maintained at a constant temperature of 20 ± 2 °C and a relative humidity of 60% ± 5%. After the samples were cured for 7 days, three samples were used to measure the drying shrinkage strain, whereas the other three samples were utilized to assess the drying shrinkage water loss ratio. Notably, on the final day of curing, the sample was saturated with water for 24 h. The water loss ratio, drying shrinkage strain, and dry shrinkage coefficient of the samples can be calculated using the following equations:

(6) $$w_{i}=\left(m_{i}-m_{i+1}\right)/m_{p}$$

(7) $$\varepsilon_{i}=\delta_{i}/l$$

(8) $$\alpha_{di}=\varepsilon_{i}/w_{i}$$

(9) $$\alpha_{d}=\sum \varepsilon_{i}/\sum w_{i}$$

where *w_i_* is the water loss ratio measured at the *i*-th (%); *m_i_* is the weighing quality measured at the *i*-th (g); *m_p_* is the mass of the sample after drying (g); *l* is the length of the sample (mm); *δ_i_* is the drying shrinkage measured at the *i*-th (mm); *ε_i_* is the drying shrinkage strain measured at the *i*-th (%); *α_di_* is the drying shrinkage strain measured at the *i*-th (%); and *α_d_* is the total dry shrinkage coefficient (%).

#### 2.3.9. Test Method for Temperature Shrinkage

The test method for temperature shrinkage was measured according to the T0855-2024 method of JTG 3441-2024 [38]. The curing age of the sample was 7 days. On the final day of curing, the sample was saturated with water for 24 h. Next, the sample was placed in an oven at 105 ± 1 °C for 10–12 h to achieve a constant weight. After drying, the sample was stored in a dry, ventilated area until it reached room temperature. The samples were tested using the sensor method. The temperature shrinkage strain and temperature shrinkage coefficient of the samples can be calculated using the following equation:

(10) $$\varepsilon_{i}=\delta_{i}/l$$

(11) $$\alpha_{ti}=\varepsilon_{i}/(T_{i}-T_{i+1})$$

where *δ_i_* is the temperature shrinkage measured at the *i*-th (mm), *l* is the length of the sample (mm), *ε_i_* is the temperature shrinkage strain measured at the *i*-th (%), *T_i_* is the temperature range at the *i*-th (°C), and *α_ti_* is the temperature shrinkage coefficient measured at the *i*-th (%).

#### 2.3.10. Test Methods for Minerals and Microstructures

Before the mineralogy and microstructure were analyzed, representative fragments from mixed cementitious materials cured for 28 days were lyophilized in a freeze dryer at −30 °C for 48 h. To investigate the mineralogical composition, the freeze-dried fragments were ground and sieved through a 200-mesh sieve (0.074 mm) prior to scanning for 20 min using a Rigaku SmartLab SE X-ray diffractometer equipped with a Cu Kα source, covering a range from 10° to 80° with a step size of 0.02°. The X-ray diffractometer used in this study is manufactured by Rigaku Corporation, which is located in Tokyo Metropolis, Japan. By analyzing the diffraction patterns, the diffraction peaks were identified and analyzed using the software Jade6 to determine the presence of various mineral phases in the sample. The pore structure was analyzed using an AutoPore IV 9500 automatic mercury porosimeter, which operated within a pressure range of 0.2–33,000 psi. The AutoPore IV 9500 automatic mercury porosimeter used in this study is manufactured by Micromeritics Instrument (Shanghai) Ltd., which is located in Shanghai, China. The freeze-dried fragments were then sputter-coated with a thin layer of gold and analyzed using a Sigma 300 instrument to identify the micromorphology and hydrated phases. The Sigma 300 instrument used in this study is manufactured by Carl Zeiss AG, which is located in Oberkochen, Germany.

**Figure 5:**
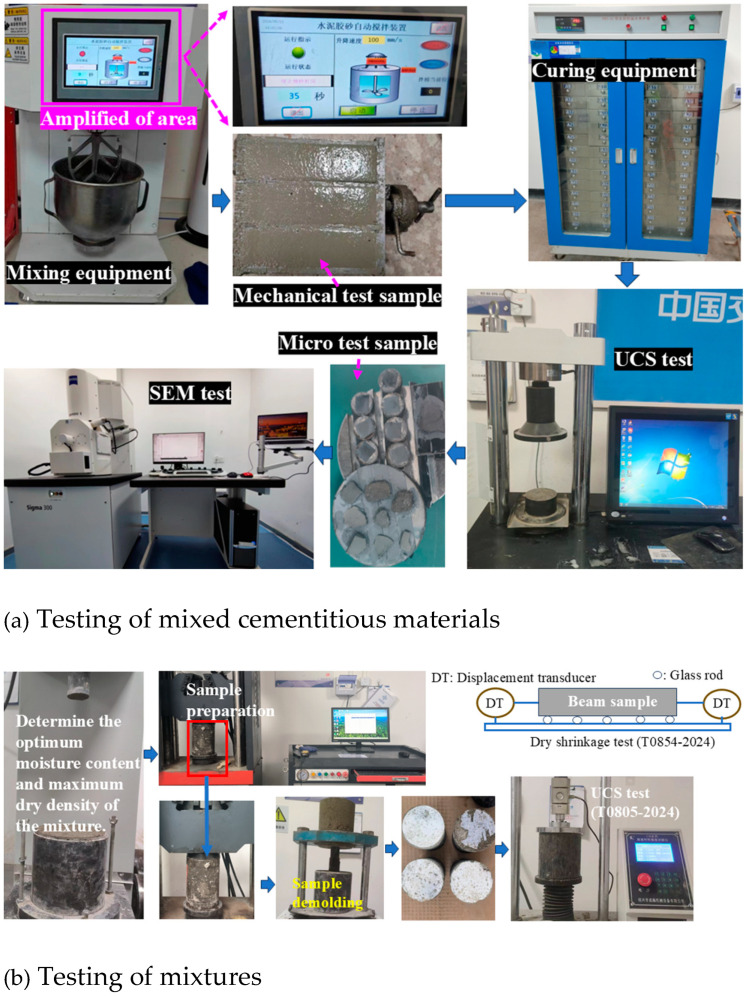
Test process.

The test process is shown in Figure 5.

## 3. Results

### 3.1. Mixed Cementitious Materials

#### 3.1.1. Compressive Strength

**Figure 6:**
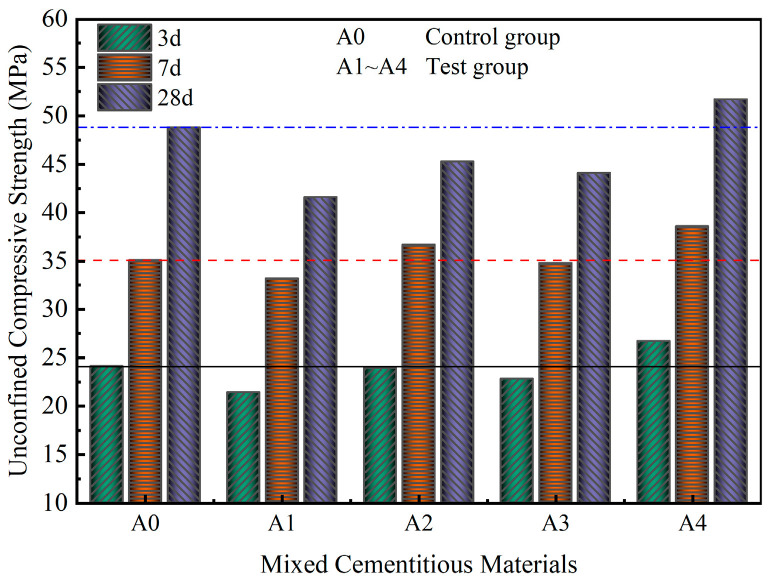
UCSs of mixed cementitious materials.

The effect of industrial solid waste on the compressive strength of mixed cementitious materials is illustrated in Figure 6. As shown in Figure 6, after curing for 3 days, the UCS of A0 was 24.1 MPa, which was higher than those of A1, A2, and A3. Notably, A4 demonstrated a UCS of 26.7 MPa, exceeding that of the control group A0. With the continued hydration of the mixed cementitious materials, after curing for 7 days, the UCSs of A2 (36.7 MPa) and A4 (38.6 MPa) surpassed that of A0 (35.1 MPa), whereas the UCSs of A1 and A3 remained lower than that of A0. After curing for 28 days, the UCSs were ranked in the following order: A4 > A0 > A2 > A3 > A1, with A4 exhibiting a 5.9% increase in UCS compared with A0.

At the curing times of 3, 7, and 28 days, the UCS of A2 was greater than those of A1 and A3 but lower than those of A0 and A4. Both GGBS and SS contained silico–aluminous compounds, with the reactivity of GGBS significantly surpassing that of SS [14]. In the alkaline environment created by OPC, samples undergo hydration reactions, resulting in the generation of substantial heat, which leads to a rapid increase in the internal temperature of the mixed cementitious materials. A2 produced a greater quantity of gel substances in a shorter time frame than A1, demonstrating that A2 presented a higher UCS than A1. The UCS of A1 indicated that OPC had a limited ability to activate the reactivity of SS. In contrast, the inclusion of FGD in A4, alongside OPC, enhanced the reactivity of both SS and GGBS [14]. This synergy enabled the formation of a greater variety of hydration products, and an increased quantity of these products was advantageous for improving the UCS. The physical filling effects of SS, GGBS, and FDG with varying particle sizes in A4 contributed to a denser structure, which was also one of the factors influencing the increase in UCS of A4.

#### 3.1.2. pH Value

**Figure 7:**
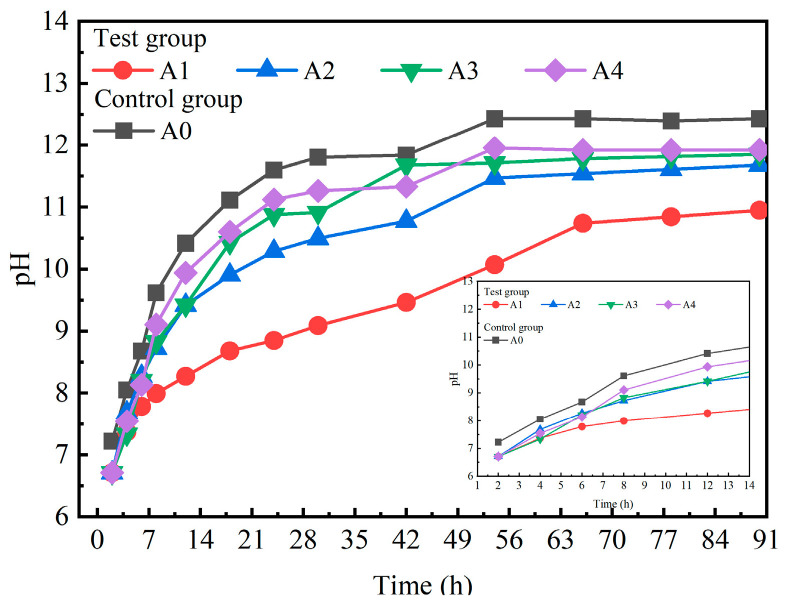
pH evolution of mixed cementitious materials.

Figure 7 shows the evolution of the pH of the pore mixture across the test groups and the control group.

The control group A0 clearly maintained consistently higher pH values than the test groups A1, A2, A3 and A4, with A1 exhibiting the lowest pH values among all the measurements. Furthermore, the pH values of test groups A1, A2, A3, and A4 closely resembled that of the control group A0. For A0, the OPC contained large amounts of C_2_S and C_3_S, which hydrated to produce Ca(OH)_2_, resulting in an abundance of OH^−^ in the pore mixture. When SS and GGBS were in contact with water, the mineral phases on the surface, such as CaO, C_2_S, and C_3_S, began to dissolve [14]. This process released significant amounts of Ca^2+^ and OH^−^, along with smaller quantities of Al^3+^ and Si^4+^, resulting in an increase in the pH of the system. As the hydration reaction progressed, the concentration of Ca^2+^ in the system gradually increased. This Ca^2+^ began to combine with OH^−^ in the water, leading to the precipitation of Ca(OH)_2_ crystals. Moreover, due to the lower amounts of C_2_S and C_3_S in the SS, some of the Ca(OH)_2_ crystals reacted with the silicate and aluminate minerals present in the SS to form C-S-H and C-A-H gels [39,40]. In contrast, the higher contents of active Al_2_O_3_ and SiO_2_ in GGBS resulted in a greater pH value in A2 than in A1. With the diffusion of hydration products, such as Ca(OH)_2_ crystals and C-S-H gel, more reaction sites were exposed in the system [41]. This increased exposure facilitated the dissolution of more mineral phases, leading to the release of Ca^2+^ and OH^−^. Consequently, the concentration of OH^−^ in the system increased. A4 consisted of OPC, FGD, GGBS, and SS. The SO_4_^2−^ provided by FGD reacted with the C-S-H gel in a reverse hydration process, leading to the formation of ettringite crystals [42]. The higher solubility of FGD served as a catalyst, promoting the generation of hydration products [14]. Hydration products, including C-S-H gel and ettringite crystals, diffused among the unreacted particles, resulting in the gradual stabilization of the pore mixture’s pH.

### 3.2. Seven-Day Unconfined Compressive Strength of the Mixture

**Figure 8:**
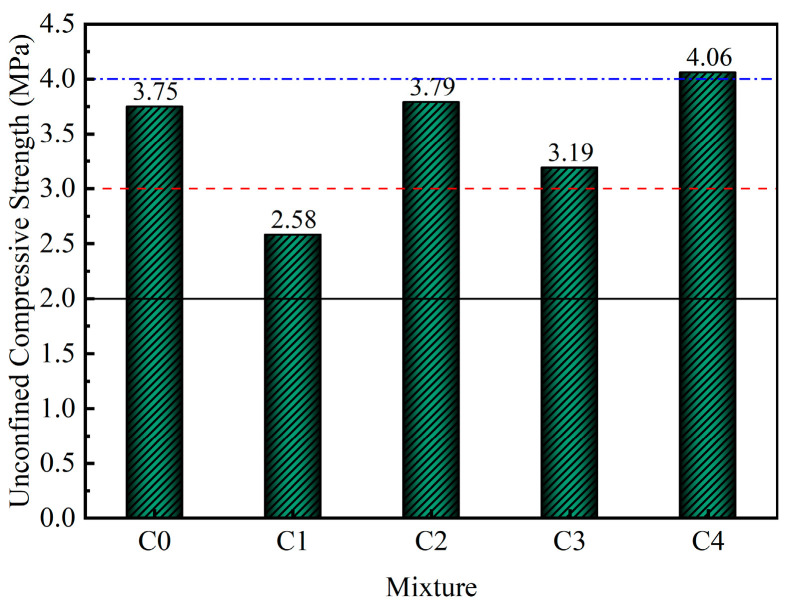
Effects of mixed cementitious materials on the unconfined compressive strength of the mixture at 7 d.

The 7-day unconfined compressive strength is often used as an important design parameter to determine whether a material meets the strength requirements of a project. The effects of different mixed cementitious materials on the 7-day UCS of the mixture are shown in Figure 8.

As illustrated in Figure 8, the UCSs of C2 (3.79 MPa) and C4 (4.06 MPa) were greater than that of the control group C0 (3.75 MPa), whereas the UCSs of C1 (2.58 MPa) and C3 (3.19 MPa) were lower than that of C0. According to the Chinese standard JTG D50-2017 [43], the UCS values for C0, C1, C2, C3, and C4 exceeded 2.0 MPa, thereby meeting the requirements for secondary highway bases. Furthermore, the UCS values for C2, C3, and C4 surpassed 3.0 MPa, satisfying the criteria for first-class highway bases. Notably, the UCS of C4 exceeded 4.0 MPa, fulfilling the requirements for expressway base layers. Thus, by adjusting the type of cementitious material in the mixture, the UCS could be tailored to meet the specifications for various road grade base layers.

### 3.3. Recycled Concrete Structural Material

#### 3.3.1. UCS and STS of the Mixture

**Figure 9:**
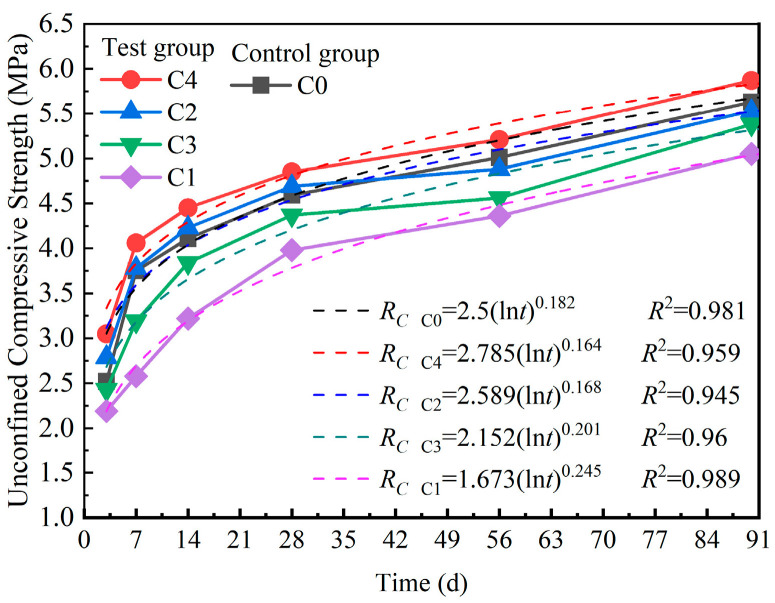
UCS curves of the mixtures.

**Figure 10:**
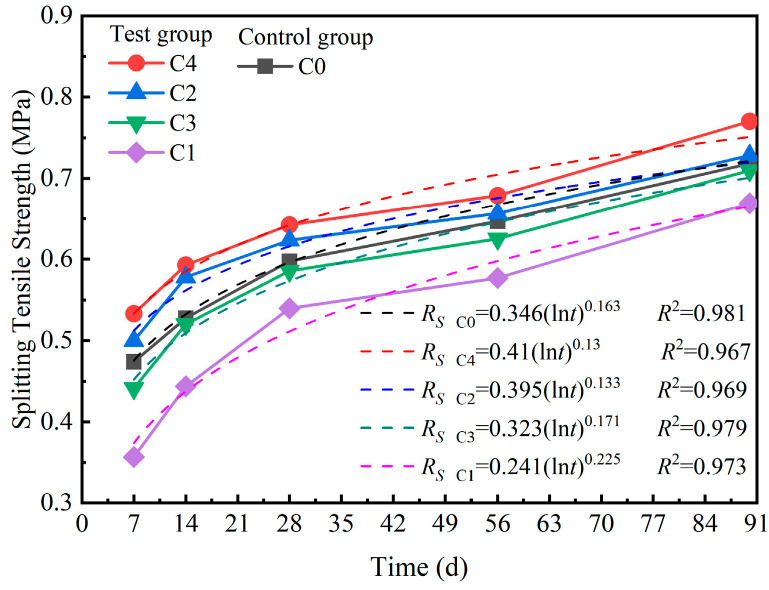
STS curves of the mixtures.

Figure 9 and Figure 10 illustrate the effects of different cementing materials on the UCS and STS of the mixture. As shown in Figure 9, from 3 days to 28 days, the UCS of C4 consistently exceeded those of C0, C1, and C3. The UCS of C2 was greater than that of the control group C0, whereas the UCSs of C1 and C3 were lower than that of C0. From 28 days to 90 days, only the UCS of C4 remained higher than that of C0, with the UCSs of C1, C2, and C3 falling below that of C0. Figure 10 shows that from 7 days to 90 days, the STSs of C4 and C2 consistently exceeded that of the control group C0, whereas the STSs of C1 and C3 were lower than that of C0. Specifically, the STS of C4 increased by 7.1–12.5% compared with that of C0, whereas the STS of C0 increased by only 1.4–7.3% compared with that of C3.

With a consistent dosage of cementing material, the UCS and STS values of C0, C1, C2, C3, and C4 tended to increase with increasing curing time. The rate of strength development from 3 days to 28 days was greater than that from 28 days to 90 days. All samples exhibited similar trends, with UCS and STS increasing nonlinearly with time, suggesting the suitability of a logarithmic function for fitting. The strength growth equations derived from the fitting analysis are presented in Figure 9 and Figure 10, with the coefficients of determination (*R*^2^) exceeding 0.95.

As listed in Table 8, the crushing values of pretreated recycled concrete aggregates, in ascending order, were B4-RCA < B2-RCA < B3-RCA < B1-RCA. Similarly, the UCS and STS of the mixtures followed the trend C4 > C2 > C3 > C1. The untreated recycled concrete aggregates exhibited numerous cracks and surface voids. However, after being soaked in cementitious materials, the cracks within the recycled concrete aggregates were filled with hydration products, which led to a reduction in surface voids. Notably, the UCS of A1 was lower than that of A0. Consequently, despite the recycled concrete aggregates in C1 being soaked in B1 solution, this treatment could not compensate for the deficiencies in hydration strength associated with the cementitious materials. As a result, the UCS of C1 was consistently lower than that of C0.

The STS of the mixed materials was influenced not only by the strength of the recycled concrete aggregates but also by the cohesion of the internal cementitious materials [24]. The surfaces of the pretreated recycled concrete aggregates were coated with gel substances, and the mixed materials C1, C2, C3, and C4 contained more gel substances than C0. The presence of these gel substances enhanced cohesion. Although the lower strength of the recycled concrete aggregate particles adversely affects the splitting tensile strength, the cohesion provided by the gel substances can partially mitigate this effect. Specifically, when the dosage of cementitious materials was 5%, the differences in the STS and UCS values between C4 and C0 were 0.03–0.07 MPa and 0.2–0.35 MPa, respectively. In contrast, the differences in the STS and UCS values between C4 and C1 were 0.04–0.11 MPa and 0.82–1.48 MPa, respectively. These results indicated that the influence of different types of mixed cementitious materials on the splitting tensile strength was less pronounced than their impact on the UCS.

The STSs of C1, C2, C3, and C4 were 0.66, 0.72, 0.71, and 0.77 MPa, respectively, after curing for 90 days. All values met the design reference value (not less than 0.4 MPa) for splitting tensile strength according to the Chinese standard JTG D50-2017 [43].

#### 3.3.2. Compressive Modulus of Resilience and Splitting Modulus of Resilience for the Mixtures

**Figure 11:**
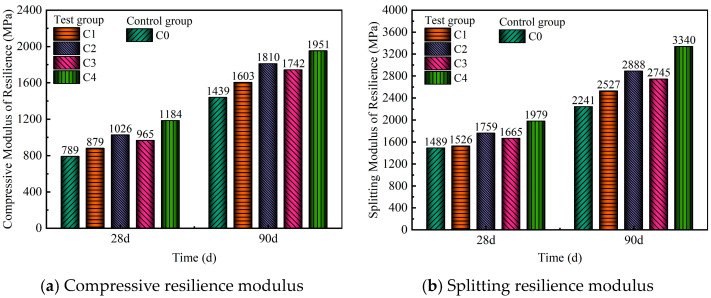
Compressive and splitting resilient moduli of the mixture.

The CRM and SRM of the mixture are presented in Figure 11a,b, respectively. Figure 11a clearly shows that the CRMs of C1, C2, C3, and C4 were greater than those of the control group C0. The order of the CRM from largest to smallest for C1, C2, C3, and C4 was C4 > C2 > C3 > C1. The CRMs of C1, C2, C3, and C4 increased with increasing curing time. According to Figure 11a,b, the SRMs of C1, C2, C3, and C4 exhibited the same trend as the CRMs. At a curing time of 28 days, the CRMs of C4 were 50%, 35%, 15%, and 23% greater than those of C0, C1, C2, and C3, respectively. After curing for 90 days, the CRMs of C4 were 36%, 22%, 8%, and 12% greater than those of C0, C1, C2, and C3, respectively. Similarly, after curing for 28 days, the SRM of C4 was 33%, 30%, 13%, and 19% greater than those of C0, C1, C2, and C3, respectively, and at a curing time of 90 days, the SRM of C4 was 49%, 32%, 16%, and 22% greater than those of C0, C1, C2, and C3, respectively. The CRMs of C1, C2, C3, and C4 increased at a greater rate from 0 to 28 days than from 28 to 90 days, whereas the SRM increased at a lower rate from 0 to 28 days than from 28 to 90 days. Additionally, the SRMs of C1, C2, C3, and C4 increased at higher rates from 0 to 28 days than from 28 to 90 days. However, C1, C2, C3, and C4 met the design reference values (not less than 1300 MPa) for the compressive resilience modulus, as specified by the Chinese Standard JTG D50-2017. The CRMs of C1, C2, C3, and C4 met the specifications only at a curing time of 90 days. Conversely, the CRMs of C1, C2, C3, and C4 failed to meet the specifications at a curing time of 28 days.

The stiffness of stabilized materials depends on the modulus of the material, the modulus of the reaction products, and the structural configuration [44,45]. The lower compressive resilience modulus of the materials resulted in a greater deformation of the structures when subjected to loading. Consequently, C0 induced greater deformation in the structures when subjected to loading than C4 induced. Due to the use of similarly graded aggregates, the compressive resilience modulus of the RCSM depended on the hydration of the mixed cementitious materials (A1, A2, A3, and A4) and the pretreated recycled concrete aggregate (B1-RCA, B2-RCA, B3-RCA, and B4-RCA). Compared with those of C1, C2, C3, and C4, the hydration products produced by the hydration reaction in C0 decreased. C1, C2, C3, and C4 contained more hydration products than C0 contained, resulting in higher CRMs and SRMs for C1, C2, C3, and C4 than for C0.

### 3.4. Drying Shrinkage and Temperature Shrinkage Properties of the Mixture

**Figure 12:**
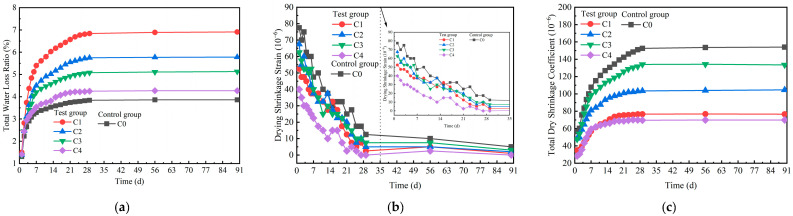
Drying shrinkage test results of the mixture. (**a**) Relationship between total water loss ratio and time; (**b**) relationship between drying shrinkage strain and time; (**c**) relationship between total dry shrinkage coefficient and time.

The total water loss ratio, drying shrinkage strain, and total dry shrinkage coefficient of the mixture are shown in Figure 12a–c, respectively. As illustrated in Figure 12a, the total water loss rates of C1, C2, C3, and C4 rapidly increased from 0 to 10 days. Therefore, in the first 10 days after construction, attention was given to the curing conditions. The loss of water was caused mainly by the hydration of the mixture. From 10 to 30 days, the total water loss rates of these samples increased at a slower rate. From 30 to 90 days, the total water loss rates of C1, C2, C3, and C4 stabilized. The control group C0 and the test groups presented similar trends in terms of total water loss rates. At 90 days, the total water loss rates for C0, C1, C2, C3, and C4 were 3.86%, 6.91%, 5.78%, 5.13%, and 4.27%, respectively. Notably, C1 presented the highest total water loss rate of 6.91%, whereas the control group C0 presented the lowest rate of 3.86%. C1, C2, C3, and C4 all contained industrial solid waste. The different specific surface areas of SS, GGBS, and FDG resulted in more free water being absorbed by C1, C2, C3, and C4 in the saturated state than by C0 [46]. Figure 12b shows that the drying shrinkage strains of C0, C1, C2, C3, and C4 all decreased with time from 0 to 30 days. The drying shrinkage strains of C0, C1, C2, C3, and C4 tended to be stable from 30 to 90 days. The drying shrinkage strains of C1, C2, C3, and C4 were lower than those of the control group C0. As shown in Figure 12c, the total dry shrinkage coefficients of C0, C1, C2, C3, and C4 were 154 × 10^−6^, 76.61 × 10^−6^, 104.67 × 10^−6^, 133.5 × 10^−6^, and 69.86 × 10^−6^, indicating reductions of 50.3%, 32%, 13.3%, and 54.6% for C1, C2, C3, and C4, respectively, compared with those of C0. The total dry shrinkage coefficients of C1, C2, C3, and C4 were lower than those of the control group C0. SS, GGBS, and FDG were used as supplementary cementitious materials. The addition of supplementary cementitious materials can have different effects on drying shrinkage based on their reactivity and influence on hydration. This can be related to the reactivity of SS, GGBS, and FDG, which can change the hydration and increase the self-desiccation of the matrix [47,48,49]. Due to the hydration reaction of cement with SS, GGBS, and FDG, it provides a robust bonding force for the matrix, enhancing the integrity of the aggregates. When shrinkage stresses occur between the internal skeleton due to water loss, the stronger bonding force can more effectively resist deformation caused by shrinkage stresses, thus reducing drying shrinkage [50]. In addition, the hydration reactions of f-CaO and f-MgO in SS and CaSO_4_·2H_2_O in FDG produce hydration products that exhibit volume expansion, facilitating the reduction in shrinkage in the mixture [51].

**Figure 13:**
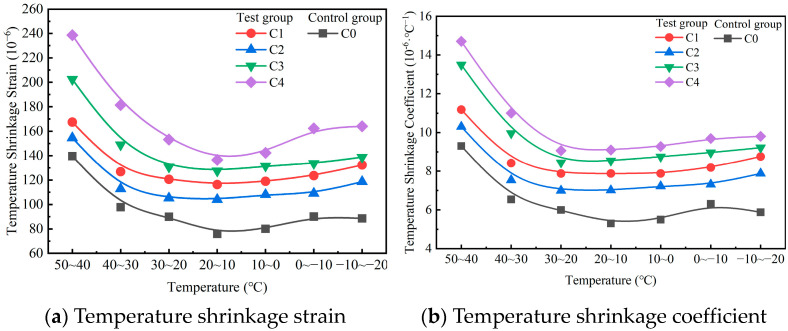
Temperature shrinkage test results of the mixture.

The temperature shrinkage strain and temperature shrinkage coefficient of the mixture are presented in Figure 13a,b, respectively. Figure 13 shows that the temperature shrinkage coefficients of C0, C1, C2, C3, and C4 exhibited variation trends similar to those of the temperature shrinkage strain. Specifically, both the temperature shrinkage coefficient and the temperature shrinkage strain first decreased but then slightly increased within the temperature range of −20–50 °C. The temperature shrinkage coefficient and temperature shrinkage strain of C4 were greater than those of the control group C0. C4 contained many types of cementing materials, including OPC, SS, GGBS, and FDG. Hydration reactions occurred among cementing materials, resulting in a wide variety of hydration products. Only OPC was present in C0 as a cementing material, which made the hydration products relatively simple. Different types of hydration products are sensitive to temperature changes [47]. Because C4 contained more types of hydration products, the temperature shrinkage coefficient and temperature shrinkage strain of C4 were greater.

The average temperature shrinkage coefficients of C0, C1, C2, C3, and C4 were 8.97 × 10^−6^·°C^−1^, 12.04 × 10^−6^·°C^−1^, 10.85 × 10^−6^·°C^−1^, 13.47 × 10^−6^·°C^−1^, and 14.53 × 10^−6^·°C^−1^, respectively. The average temperature shrinkage coefficient of C4 was 14.53 × 10^−6^·°C^−1^, which was higher than the 8.97 × 10^−6^·°C^−1^ of C0. As shown in Figure 12c, the decrease in the dry shrinkage coefficient of C4 was 70.09 × 10^−6^. The decrease in the total dry shrinkage coefficient of C4 was greater than the increase in the average temperature shrinkage coefficient. When the required strength target was reached, the use of C4 avoided the risk of dry shrinkage cracking in the pavement base and reduced the formation of pavement cracks caused by base cracking. To increase the durability of the road surface, the road use time was extended.

### 3.5. Freeze–Thaw Resistance of the Mixture

**Figure 14:**
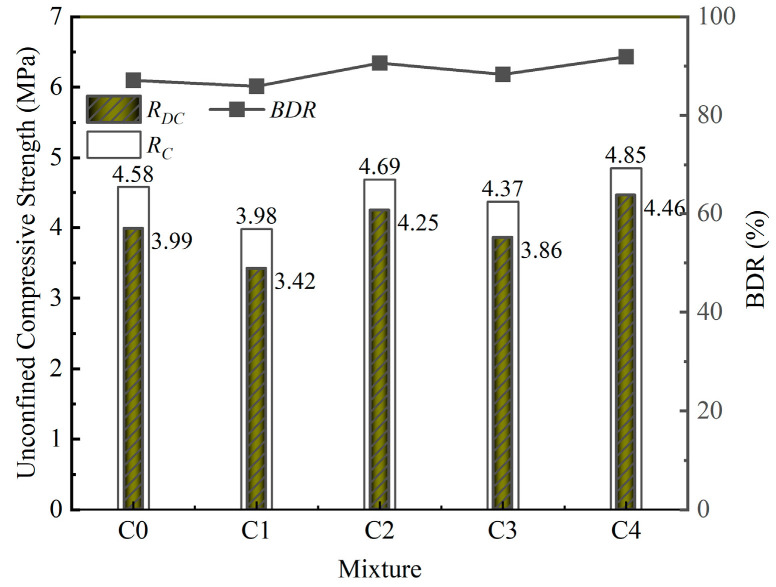
Freeze–thaw test results of the mixture.

The freeze–thaw resistance of the mixture is illustrated in Figure 14. As shown in Figure 14, the UCSs of C0, C1, C2, C3, and C4 all decreased after five freeze–thaw cycles. The BDRs of C0, C1, C2, C3, and C4 were similar, ranging from 85.9 to 91.9%. The residual compressive strength of recycled cement-stabilized macadam after 28 days of curing under five freeze–thaw cycles should not be less than 50%, which represents the anti-freezing index for recycled cement-stabilized macadam in medium–heavy frost regions [44]. The frost resistance of the pavement base is influenced primarily by the internal pore distribution and the water absorption of the aggregates [52]. The internal pore distribution of the mixture was related to the degree of hydration of the cementitious material. Compared with those of C0, the hydration products of C1, C2, C3, and C4 were more abundant, which facilitated the bonding of the particles. The BDR of C1 was small, which was related to the water absorption of the aggregate and the bonding between the gelling materials. The above results indicate that C1 contained relatively more water, and the ice crystal volume generated during the freezing process was greater. The bonding effect of the SS-assisted OPC was worse than that of the pure OPC; therefore, the freezing resistance of C1 was not as good as that of C0.

## 4. Microscopic Test Results of Mixed Cementitious Materials

### 4.1. X-Ray Diffraction

**Figure 15:**
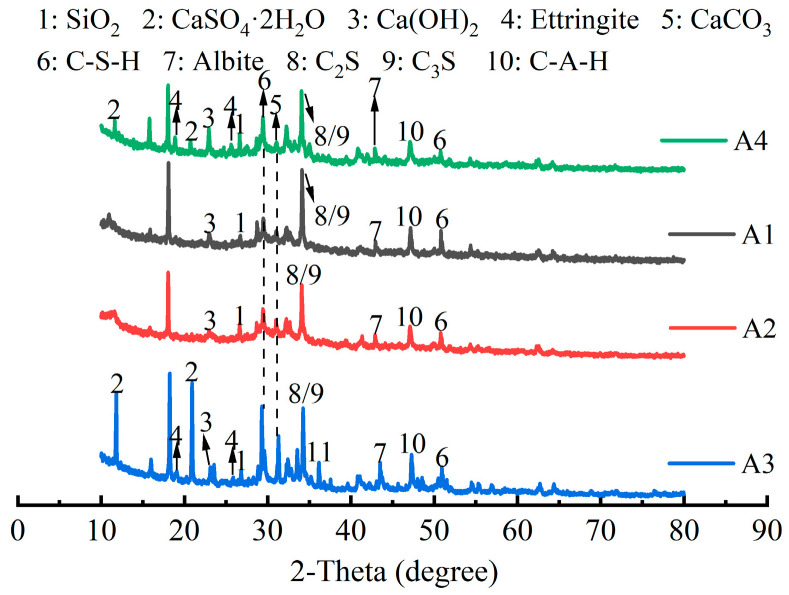
XRD analysis of mixed cementitious materials.

The XRD patterns of different mixed cementitious materials are shown in Figure 15. The diffraction peaks of all the samples revealed the presence of C-S-H gel (2*θ* = 29.5°, 2*θ* = 50.8°), C-A-H gel (2*θ* = 47.1°), and incompletely hydrated C_3_S and C_2_S (2*θ* = 34.1°). However, gypsum crystals (2*θ* = 11.6°, 2*θ* = 20.7°) and ettringite crystals (2*θ* = 18.9°, 2*θ* = 25.6°) also existed in A3 and A4. In A1, the hydration induction period of the OPC was shorter than that of the SS. OPC hydrated before SS. OH^−^ and Ca^2+^ were released continuously during the OPC hydration process, resulting in an increase in the alkaline environment of the matrix, which provided a suitable alkaline environment for activating the SS. Under the action of OH^−^, the hydrated product of SS was a C-S-H gel [53,54]. In A2, the activity of GGBS was relatively high. Silicon existed as a SiO_4_ tetrahedron in GGBS, and aluminum existed as an AlO_4_ tetrahedron in GGBS. The reaction of soluble Ca^2+^ with GGBS promoted the dissociation of silicon (aluminum) oxygen tetrahedrons to form C-S-H gel [14]. In A3, FDG contained CaSO_4_, CaSO_4_·0.5H_2_O, and CaSO_4_·2H_2_O [55]. The hydration of OPC produced C-A-H gel (C_4_AH_13_), whereas CaSO_4_·2H_2_O in FDG and C_4_AH_13_ in OPC formed ettringite crystals. In A4, the hydration products of the OPC were Ca(OH)_2_ (2*θ* = 22.9°), C-S-H gel, and C-A-H gel. Moreover, FDG provided Ca^2+^ and SO_4_^2−^ to the matrix. The ions produced by the hydration of FDG helped stimulate the hydration of GGBS and SS. SS was hydrated to produce Ca(OH)_2_ under the excitation of FDG, which provided an alkaline environment for the entire matrix. GGBS generated C-S-H gel and ettringite crystals under the excitation of FDG and an alkaline environment [56,57]. SiO_2_ (2*θ* = 26.7°) and albite (2*θ* = 42.9°) were present in A1, A2, A3, and A4. Due to the presence of inert components (RO phase) and low-activity C_2_F in A1 [13], the performance of A1 was worse than that of A0, as reflected by the mechanical strength.

### 4.2. Scanning Electron Microscopy

**Figure 16:**
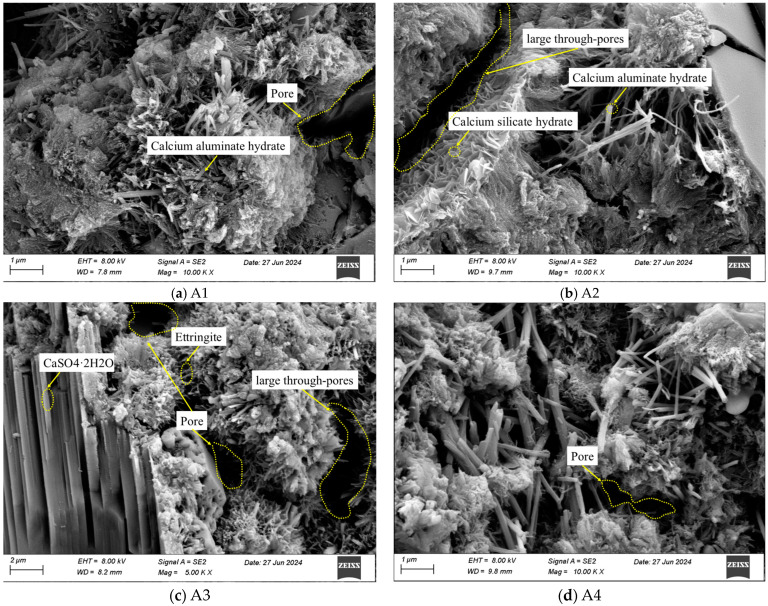
SEM images of (**a**) A1, (**b**) A2, (**c**) A3, and (**d**) A4.

The microscopic morphologies of the mixed cementitious materials are shown in Figure 16. Figure 16 shows that the hydration products of A1 and A2 were mainly flocculent C-S-H gel and fibrous C-A-H gel, whereas the hydration products of A3 and A4 included tabular gypsum crystals and needle-like ettringite crystals [12,58]. The SEM and XRD results were similar. No laminated Ca(OH)_2_ was found in A1, A2, A3, or A4, which may have been due to the consumption of Ca(OH)_2_ in the hydration process. In Figure 16a, a large area of pores was observed after the hydration of the A1 cementitious material, and the gel substances were mainly distributed in clusters, resulting in an uneven pore distribution and a limited bonding effect of the particles. The bonding performance of the A1 cementitious material was insufficient, which was consistent with the low strength of C1 obtained in the mechanical test (Figure 9). As shown in Figure 16b, the large through-pores were observed after the hydration of the A2 cementitious material. Although there was flocculent C-S-H gel between large through-pores, no effective connections formed between the particles. Figure 16c shows that after the hydration of the A3 cementitious material, a small amount of gel substances filled the pores. However, the large through-pores and smaller pores were still observed. As shown in Figure 16d, after the hydration of the A4 cementitious material, the large through-pores and pores were filled with gel substances and crystals. The fibrous gel intertwined with the needle-like crystals to form a network structure, making the structure denser [59]. This was consistent with the higher strengths of A4 and C4, as indicated by the mechanical test results.

### 4.3. Mercury Intrusion Porosimetry

**Figure 17:**
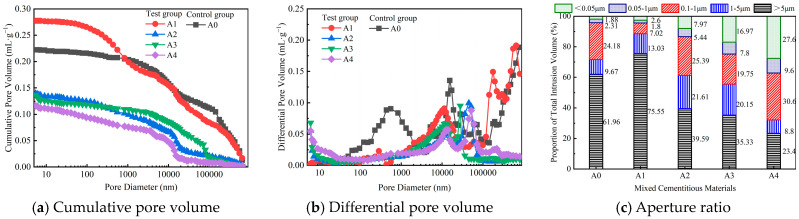
Mercury intrusion porosimetry test results of mixed cementitious materials.

The cumulative pore volume, differential pore volume, and aperture ratio of the mixed cementitious materials are shown in Figure 17a–c, respectively. According to Figure 17a, the cumulative pore volume of A1 (0.277 mL/g) was greater than that of the control group A0 (0.222 mL/g), whereas the cumulative pore volumes of A2, A3, and A4 (0.139 mL/g, 0.136 mL/g, and 0.116 mL/g, respectively) were lower than those of A0. Due to the differences in cumulative pore volume, it was speculated that the degrees of hydration reaction of the different binding materials had significant effects on the strength performance. The cumulative pore volume of A1 was greater, whereas the cumulative pore volume of A4 was lower. This was consistent with the changes in the mechanical strength of the corresponding mixed cementitious materials and mixtures. According to Figure 17b, the differential pore size curves of A0 and A1 showed a distribution of three peaks, whereas the differential pore size curves of A2 and A4 showed a bimodal distribution. In the range of 0.2–1.6 μm, the pore sizes of A1, A2, A3, and A4 decreased compared with those of the control group A0. The reduction in aperture may have been due to the filling effect of industrial solid waste particles. In the range of 6–20 μm, the peaks of A1, A2, A3, and A4 all shifted to the left compared with those of the control group A0. The pore diameter changed from approximately 15 μm to approximately 11 μm, resulting in an optimized pore structure. There were relatively more pores larger than 65 μm in A0 and A1, whereas A2, A3, and A4 had relatively more pores smaller than 0.01 μm. According to Figure 17c, compared with A0, the proportion of pore sizes greater than 5 μm in A2, A3, and A4 decreased, whereas the proportions of pore sizes ranging from 1 to 5 μm and from 0.05 to 1 μm increased. In addition, the proportion of pore diameters greater than 5 μm in A4 was relatively small, and the pore size distribution across various ranges was relatively more uniform, which allowed A4 to exhibit greater mechanical properties. In A1, the percentage of pores larger than 5 μm was greater, reaching 75.55%, whereas the percentage of pores smaller than 1 μm was only 3.4%, indicating the unevenness of the pore size distribution.

## 5. Discussion

### 5.1. Influence of Mixed Cementitious Materials on the Macroscopic Properties of Recycled Concrete Aggregates

Compared with NA, RCA presented higher water absorption rates and greater crushing values. When the aggregate particle size ranged from 9.5 to 19 mm, the water absorption rates of the NA and RCA were 0.95% and 4.78%, respectively, whereas their crushing values were 8.9% and 25.32%, respectively. The water absorption rate and density of RCA are influenced by the water–cement ratio of the original concrete and the quantity of adhered mortar [60]. The RCA differed from the NA primarily because of the presence of two additional components: the adhered mortar and the interfacial transition zone between the original NA and the original cement mortar. The porosity and cracks at the interface are greater than the porosity and cracks of the hardened cement mortar on the surface of the recycled aggregate particles [61]. The characteristics of the initial interface area depend on the quality of the mortar rather than the quantity of mortar adhesion [62].

A mixed solution consisting of OPC, SS, GGBS, and FDG was used to improve the performance of RCA. The mixed solution containing active substances can effectively fill the pores and voids within the adhesive mortar and react with the calcium hydroxide present on the surface of the RCA, forming a C-S-H gel [28]. Furthermore, the newly composed mixed solution can completely soak the RCA, creating a thin layer of paste on the surface of the RCA. This paste layer can be uniformly distributed around the RCA particles, serving as a protective film and enhancing the bonding quality of the mortar. The crushing value of the RCA after soaking in mixed solution decreased from 25.32% to 15.79%.

### 5.2. Microscopic Mechanisms of Action in Mixed Cementitious Materials

C4 exhibited high strength and good shrinkage performance. This phenomenon primarily arose from the synergistic effects of the mixed cementitious materials. The hydration reaction of the OPC produced C-S-H gel, C-A-H gel (Equation (12)) [63,64], and Ca(OH)_2_ crystals. In the system of SS, GGBS, FDG, and OPC, under the activation of FDG, GGBS first underwent a hydration reaction and then continued to dissociate. The Ca–O bond in the GGBS particles was the first to break, followed by the Mg–O bond and, finally, the Al–O bond. Ca^2+^ or Mg^2+^ dissociated from the surface of the GGBS particles into the liquid phase, and the surface of the GGBS particles had a negative charge [9]. To maintain charge balance, the water was ionized, and the ionized hydrogen ions (H^+^) were adsorbed to the surface of the GGBS particles to achieve charge balance. The adsorbed hydrogen ions combined with the broken Si–O bonds on the surface of the GGBS particles to form -Si–OH, and the structure of the silica–oxygen tetrahedron was continuously destroyed. As the hydration reaction of the GGBS continued, the concentration of OH^−^ in the solution increased, and the pH gradually increased. The solubility of silicon oxides also increased, resulting in continuous increases in the concentrations of H_2_SiO_4_^2−^, HSiO_4_^3−^, and SiO_4_^4−^ in the solution [65], while the Al–O bonds in the GGBS also broke [14,66,67,68]. These ions reacted with OH^−^ and Ca^2+^ to form C-S-H gel.

The bonding structure of silicate and aluminate minerals in SS primarily consisted of silicon–oxygen and aluminum–oxygen bonds, manifested as SiO_4_ tetrahedrons and AlO_4_ tetrahedrons or AlO_6_-coordinated polyhedrals [12]. FDG was used as an activator to stimulate the activity of SS, releasing significant amounts of OH^−^, Ca^2+^, silicon solute, and aluminum solute. This specifically included OH^−^, Ca^2+^, and SO_4_^2−^, [Al(OH)_6_]^3−^. The recombination of these ions led to the continuous breaking of the silicon–oxygen and aluminum–oxygen bonds in the SS. The steel slag also continued to dissolve. During this process, the hydration reaction produced needle- and rod-shaped ettringite crystals interglued with the C-S-H gel [13,14]. During the hydration process, the formation of ettringite crystals simultaneously disrupted the dissolution equilibrium of the glass phase in the GGBS, causing the Si–O bonds of the silicate tetrahedron in the slag to dissociate and generate H_2_SiO_4_^2−^, which then reacted with Ca^2+^ in an alkaline solution to form C-S-H gel [69]. FDG can also react with the C-A-H (C_4_AH_13_) gel generated from OPC hydration to form ettringite crystals (Equation (13)) [70].

(12) $$3CaO\cdot Al_{2}O_{3}+Ca{\left(OH\right)}_{2}+12H_{2}O\to C_{4}AH_{13}$$

(13) $$C_{4}AH_{13}+3(CaSO_{4}\cdot 2H_{2}O)+14H_{2}O\to 3CaO\cdot Al_{2}O_{3}\cdot 3CaSO_{4}\cdot 32H_{2}O+Ca{\left(OH\right)}_{2}$$

## 6. Conclusions

Through mechanical and microscopic tests, the performances of the mixed cementitious material, pretreated RCA, and mixture were evaluated. According to the laboratory test results, the main conclusions were as follows:

(1) After curing for 3 days and 28 days, the crushing value of B4-RCA decreased by 22.35% and 37.64%, respectively, compared to that of B0-RCA. At the curing times of 3 days, 7 days, and 28 days, the compressive strength of A4 increased by 10.79%, 9.97%, and 5.94% compared to A0, respectively.
(2) The UCS of C4 was higher than 4 MPa at a curing time of 7 days. At the curing times of 7 days, 28 days, and 90 days, the UCS of C4 increased by 8.35%, 5.67%, and 4.26%, respectively, compared to that of C0. The STS and CRM of C4 were not less than 0.4 MPa and 1300 MPa, respectively, at 90 days of curing.
(3) The total water loss ratio of C4 was greater than that of C0, whereas the total dry shrinkage coefficient of C4 was lower than that of C0. When the desired strength target was reached, the use of C4 reduced the possibility of drying shrinkage cracking in the pavement base layer and decreased the generation of pavement cracks caused by base layer cracking. The BDR of C4 was greater than that of C0. All the strength residual ratios of C0 and C4 were greater than 85.0%.
(4) The hydration product of A4 was mainly flocculent C-S-H gel and needle-like ettringite crystals. After the hydration of the A4 cementitious material, the fibrous gel intertwined with the needle-like crystals to form a network structure, making the structure denser. The cumulative pore volume of A4 was lower than that of A0. In A4, the percentage of pores larger than 5 μm was smaller, reaching 23.4%, whereas the percentage of pores smaller than 1 μm was 37.2%, indicating the uniformity of the pore size distribution.

Industrial solid wastes such as SS, GGBS, and FDG account for 30% of mixed cementitious material. The replacement rate in this study was fixed at 30%. Future optimization through orthogonal testing will focus on refining the ratio between OPC and industrial solid waste to achieve higher strength in mixed cementitious materials. The maximum curing time for this test was set at 90 days. Longer curing times, such as 180 days or one year, will be considered for the next study.

## Figures and Tables

**Table 1:** Physical properties of OPC.

Fineness (%)	Setting Time (min)		Loss on Ignition (%)	Normal Consistency (%)	Density (g/cm^3^)	Specific Surface Area (m^2^/kg)
	Initial	Final				
3.1	192	372	4.5	28	3.07	351

**Table 2:** Physical properties of SS, GGBS, and FDG.

Material	Grain Size Parameter (μm)			Specific Surface Area (m^2^/kg)	Normal Consistency (%)	Apparent Specific Gravity (g/cm^3^)
	D_10_	D_50_	D_90_			
SS	1.32	9.56	40.16	526	33.2	2.38
GGBS	1.52	13.61	54.98	403	25.6	2.16
FDG	11.32	55.36	79.63	201	21.8	2.05

**Table 3:** Major chemical compositions (wt.%) of OPC, SS, GGBS, and FDG.

Material	SiO_2_	Al_2_O_3_	CaO	Fe_2_O_3_	SO_3_	K_2_O	MgO	P_2_O_5_
OPC	18.9	4.95	64.55	2.39	2.65	1.26	0.88	0.34
SS	13.86	3.99	45.76	13.69	0.45	0.03	4.39	1.49
GGBS	32.1	14.1	40.1	0.29	0.51	0.55	11.1	-
FDG	2.3	0.5	49.5	0.3	44.3	-	0.3	-

**Table 4:** Design of mixed cementitious materials.

Mixed Cementitious Materials	Mass Percentage of Binder Materials (%)			
	OPC	SS	GGBS	FDG
A0	100	/	/	/
A1	70	30	/	/
A2	70	/	30	/
A3	70	/	/	30
A4	70	10	10	10

**Table 5:** Physical properties of RCA.

Sieve Size (mm)	Bulk Specific Gravity (g/cm^3^)	Apparent Specific Gravity (g/cm^3^)	Mud Content (%)	Needle and Flake Content (%)	Crushing Value (%)
19~26.5	1.22	2.603	1.45	7.6	-
9.5~19	1.32	2.691	1.89	8.3	25.32
4.75~9.5	1.39	2.712	2.01	8.2	-

**Table 6:** Gradation of the aggregates.

Gradation	Sieve Size (mm)								
	0.075	0.3	0.6	1.18	2.36	4.75	9.5	19	26.5
Gradation limits upper	5	10	15	22	31	45	62	86	100
Gradation limits lower	2	5	8	13	22	35	53	82	100
Median gradation	3.5	7.5	11.5	17.5	26.5	40	57.5	84	100
Target gradation	3	7	11	19	28	36	59	86	100

**Table 7:** Pretreatment of recycled coarse aggregate scheme.

Sample Number	Mass Percentage of Binder Materials (%)				Water-to-Binder Materials Ratio
	OPC	SS	GGBS	FDG	
B0	/	/	/	/	/
B1	70	30	/	/	0.7
B2	70	/	30	/	
B3	70	/	/	30	
B4	70	10	10	10	

**Table 8:** Crushing value of recycled concrete aggregate after pretreatment.

Types	Recycled Concrete Aggregates	Crushing Value (%)		Water Absorption (%)	
		3d	28d	3d	28d
Untreated RCA	B0-RCA	25.32	25.32	4.78	4.78
Pretreated RCA	B1-RCA	22.56	19.36	4.43	3.45
	B2-RCA	20.78	16.89	3.04	1.38
	B3-RCA	21.88	18.26	3.19	1.82
	B4-RCA	19.66	15.79	2.31	1.18

**Table 9:** Optimum moisture content and maximum dry density of the mixture.

Mixture	Mixed Cementitious Materials	Recycled Concrete Aggregates	Maximum Dry Density (g/cm^3^)	Optimum Moisture Content (%)
C0	A0	B0-RCA	2.001	7.95
C1	A1	B1-RCA	2.129	7.78
C2	A2	B2-RCA	2.181	7.64
C3	A3	B3-RCA	2.131	7.31
C4	A4	B4-RCA	2.191	7.43

**Table 10:** Experimental design for mixture testing.

Test Project	Time (d)	Sample Size (mm)
Unconfined compressive tests	7, 14, 28, 56, 90	*Φ*150 × *h*150
Splitting tensile tests		
Compressive modulus of resilience	28, 90	
Splitting modulus of resilience		
Freeze–thaw cycle test	28	
Drying shrinkage test	7	100 × 100 × 400
Temperature shrinkage test		

## Data Availability

The original contributions presented in this study are included in the article. Further inquiries can be directed to the corresponding author.

## References

[1] Ding J., Ma S.H., Shen S., Xie Z.L., Zheng S.L., Zhang Y. (2017). Research and industrialization progress of recovering alumina from fly ash: A concise review. Waste Manag..

[2] Liu W.Z., Teng L.M., Rohani S., Qin Z.F., Zhao B., Xu C.C., Ren S., Liu Q.C., Liang B. (2021). CO_2_ mineral carbonation using industrial solid wastes: A review of recent developments. Chem. Eng. J..

[3] Li N., Lv S.W., Wang W., Guo J., Jiang P., Liu Y. (2020). Experimental investigations on the mechanical behavior of iron tailings powder with compound admixture of cement and nano-clay. Constr. Build. Mater..

[4] Cheng Y.H., Huang F., Qi S.S., Li W.C., Liu R., Li G.L. (2020). Durability of concrete incorporated with siliceous iron tailings. Constr. Build. Mater..

[5] Ma B.G., Cai L.X., Li X.G., Jian S.W. (2016). Utilization of iron tailings as substitute in autoclaved aerated concrete: Physico-mechanical and microstructure of hydration products. J. Clean. Prod..

[6] da Silva Andrade Neto J., De la Torre A.G., Paula A.K. (2021). Effects of sulfates on the hydration of Portland cement-A review. Constr. Build. Mater..

[7] Tang L., Yu Z., He Z.Y., Pei S.S. (2024). Evaluation of the workability, mechanical strength, leaching toxicity and durability of sulfate solid waste composite cementitious materials. Sustain. Chem. Pharm..

[8] Su C., Zhang J.X., Ding Y.J. (2024). Research on reactivity evaluation and micro-mechanism of various solid waste powders for alkali-activated cementitious materials. Constr. Build. Mater..

[9] Wang K., Li K., Huang X., Ni W., Zhang S. (2023). Preparation of backfill materials by solidifying municipal solid waste incineration fly ash with slag-based cementitious materials. Int. J. Environ. Sci. Technol..

[10] Kourounis S., Tsivilis S., Tsakiridis P., Papadimitriou G.D., Tsibouki Z. (2007). Properties and hydration of blended cements with steelmaking slag. Cem. Concr. Res..

[11] Golewski L.G. (2024). Determination of fracture mechanic parameters of concretes based on cement matrix enhanced by fly ash and Nano-Silica. Materials.

[12] Hao X.R., Guo Y.X. (2012). Study on desulfurized gypsum-steel slag composite gelled material mechanics properties. Appl. Mech. Mater..

[13] Zhang S.Q., Wu B., Ren Y.T., Wu Z.P., Li Q., Li K.Q., Zhang M.G., Yu J.H., Liu J.L., Ni W. (2023). The preparation process and hydration mechanism of steel slag-based ultra-fine tailing cementitious filler. Gels.

[14] Liu Z.Y., Ni W., Li Y., Ba H.J., Li N., Ju Y.J., Zhao B., Jia G.L., Hu W.T. (2021). The mechanism of hydration reaction of granulated blast furnace slag-steel slag-refining slag-desulfurization gypsum-based clinker-free cementitious materials. J. Build. Eng..

[15] Niu F.S., An Y.K., Zhang J.X., Chen W., He S.T. (2021). Synergistic excitation mechanism of CaO-SiO_2_-Al_2_O_3_-SO_3_ quaternary active cementitious system. Front. Mater..

[16] Wan X.M., Li H., Che X.P., Xu P.Z., Li C.J., Yu Q. (2023). A Study on the application of recycled concrete powder in an alkali-activated cementitious system. Processes.

[17] Xi X.Y., Zheng Y.X., Zhuo J.B., Zhang P., Golewski G.L., Du C.W. (2024). Influence of water glass modulus and alkali content on the properties of alkali-activated thermally activated recycled cement. Constr. Build. Mater..

[18] Zhang P., Li Q., Wei H. (2010). Investigation of flexural properties of cement-stabilized macadam reinforced with polypropylene fiber. J. Mater. Civ. Eng..

[19] Zheng Y.X., Zhang P., Cai Y.C., Jin Z.Q., Moshtagh E. (2019). Cracking resistance and mechanical properties of basalt fibers reinforced cement-stabilized macadam. Compos. Pt. B-Eng..

[20] Ahmed H., Tiznobaik M., Huda S.B., Islam M.S., Alam M.S. (2020). Recycled aggregate concrete from large-scale production to sustainable field application. Constr. Build. Mater..

[21] Xuan D.X., Houben L.M., Molenaar A.A.A., Shui Z.H. (2012). Mechanical properties of cement-treated aggregate material—A review. Mater. Des..

[22] You L.Y., Yan K.Z., Yue Y.F., Yu T. (2020). Comparisons of Natural and Enhanced Asphalt Mixtures Containing Recycled Cement-Stabilized Macadam as Aggregates. J. Mater. Civ. Eng..

[23] Tanvir I., Asif A., Sahadat H.M., Mohammad F. (2020). Microstructure analysis and strength characterization of recycled base and sub-base materials using scanning electron microscope. Infrastructures.

[24] You L.Y., Yue Y.F., Yan K.Z., Zhou Y.B. (2021). Characteristics of cement-stabilized macadam containing surface-treated recycled aggregates. Road Mater. Pavement Des..

[25] Liao W.Y., Sun X., Sun X., Kumar A., Sun H.F., Ma H.Y. (2019). Hydration of binary portland cement blends containing silica fume a decoupling method to estimate degrees of hydration and pozzolanic reaction. Front. Mater..

[26] Katz A. (2004). Treatments for the Improvement of Recycled Aggregate. J. Mater. Civ. Eng..

[27] Tam W.V., Tam C., Le K. (2006). Removal of cement mortar remains from recycled aggregate using pre-soaking approaches. Resour. Conserv. Recycl..

[28] Tam W.V., Gao X., Tam C. (2004). Microstructural analysis of recycled aggregate concrete produced from two-stage mixing approach. Cem. Concr. Res..

[29] (2023). Common Portland cement, General Administration of Quality Supervision.

[30] Chen Z.M., Li R., Zheng X.M., Liu J.X. (2021). Carbon sequestration of steel slag and carbonation for activating RO phase. Cem. Concr. Res..

[31] Wang Q., Yan P. (2009). Hydration properties of basic oxygen furnace steel slag. Constr. Build. Mater..

[32] Aubert J.E., Husson B., Sarramone N. (2006). Utilization of municipal solid waste incineration (MSWI) fly ash in blended cement Part 1: Processing and characterization of MSWI fly ash. J. Hazard. Mater..

[33] (2001). Standard Test Method for Compressive Strength of Hydraulic Cement Mortars.

[34] Xu B., Lothenbach B., Leemann A., Winnefeld F. (2018). Reaction mechanism of magnesium potassium phosphate cement with high magnesium-to-phosphate ratio. Cem. Concr. Res..

[35] (2019). Standard Test Methods for pH of Soils.

[36] (2024). Test Methods of Aggregates for Highway Engineering.

[37] (2015). Technical Guidelines for Construction of Highway Roadbases.

[38] (2024). Test Methods of the Materials Stabilized with Inorganic Binders for Highway Engineering.

[39] Chen H., Feng P., Ye S.X., Sun W. (2018). The coupling effect of calcium concentration and pH on early hydration of cement. Constr. Build. Mater..

[40] Xu C., Ni W., Li K., Zhang S., Xu D. (2019). Hydration mechanism and orthogonal optimisation of mix proportion for steel slag-slag-based clinker-free prefabricated concrete. Constr. Build. Mater..

[41] Sajid M., Bai C., Aamir M., You Z.X., Yan Z.M., Lv X.M. (2019). Understanding the structure and structural effects on the properties of blast furnace slag (BFS). ISIJ Int..

[42] Nguyen H., Adesanya E., Ohenoja K., Kriskova L., Pontikes Y., Kinnunen P., Illikainen M. (2019). Byproduct-based ettringite binder—A synergy between ladle slag and gypsum. Constr. Build. Mater..

[43] (2017). Specifications for Design of Highway Asphalt Pavement.

[44] Yan K., Gao F., Sun H., Ge D.D., Yang S. (2019). Effects of municipal solid waste incineration fly ash on the characterization of cement-stabilized macadam. Constr. Build. Mater..

[45] You L., Yan K., Hu Y., Liu J., Ge D.D. (2018). Spectral element method for dynamic response of transversely isotropic asphalt pavement under impact load. Road Mater. Pavement Des..

[46] Bella D.C., Wyrzykowski M., Lura P. (2017). Evaluation of the ultimate drying shrinkage of cement-based mortars with poroelastic models. Mater. Struct..

[47] Wu L., Farzadnia N., Shi C., Zhang Z.H., Wang H. (2017). Autogenous shrinkage of high performance concrete: A review. Constr. Build. Mater..

[48] Hailong Y., Radlińska A. (2016). A review and comparative study of existing shrinkage prediction models for portland and non-portland cementitious materials. Adv. Mater. Sci. Eng..

[49] Kovler K., Zhutovsky S. (2006). Overview and future trends of shrinkage research. Mater. Struct..

[50] Sun X., Wu S., Yang J., Yang R.C. (2020). Mechanical properties and crack resistance of crumb rubber modified cement-stabilized macadam. Constr. Build. Mater..

[51] Suk B.C., Cheol Y.C. (2018). Hydration properties of STS-refining slag-blended blast furnace slag cement. Adv. Mater. Sci. Eng..

[52] Setzer J.M. (2001). Micro-ice-lens formation in porous solid. J. Colloid Interface Sci..

[53] Duan S., Liao H.Q., Cheng F.Q., Song H.Q., Yang H.Q. (2018). Investigation into the synergistic effects in hydrated gelling systems containing fly ash, desulfurization gypsum and steel slag. Constr. Build. Mater..

[54] Yoon S., Choi W., Jeon C. (2022). Hydration properties of mixed cement containing ground-granulated blast-furnace slag and expansive admixture. J. Mater. Cycles Waste Manag..

[55] Li H.X., Zhang H., Li L., Ren Q., Yang X.J., Jiang Z.W., Zhang Z.L. (2019). Utilization of low-quality desulfurized ash from semi-dry flue gas desulfurization by mixing with hemihydrate gypsum. Fuel.

[56] Duan S.Y., Wu H., Zhang K., Liao H.Q., Ma Z.B., Cheng F.Q. (2022). Effect of curing temperature on the reaction kinetics of cementitious steel slag-fly ash-desulfurized gypsum composites system. J. Build. Eng..

[57] Zhang M.G., Li K.Q., Ni W., Zhang S., Liu Z., Wang K., Wei X., Yu Y. (2022). Preparation of mine back filling from steel slag-based non-clinker combined with ultra-fine tailing. Constr. Build. Mater..

[58] Ni W., Li Y., Xu C.W., Xu D., Jiang Y.Q. (2019). Hydration mechanism of blast furnace slag-reduction slag based solid waste cementing materials. J. Cent. South Univ..

[59] Deng Y., Wang X., Zhou B., Xu X.J., Chen L. (2024). Systematic assessment of a multi–solid waste cementitious material: Feasibility and environmental impact. Constr. Build. Mater..

[60] Etxeberria M., Vázquez E., Marí A., Barra M. (2007). Influence of amount of recycled coarse aggregates and production process on properties of recycled aggregate concrete. Cem. Concr. Res..

[61] Zhang J.K., Taylor P.C., Shi C.J. (2015). Investigation of approaches for improving interfacial transition zone-related freezing-and-thawing resistance in concrete pavements. ACI Mater. J..

[62] Jia Z.J., Cao R.L., Zhang S.Q., Chen C., Zhang Y.M. (2023). Insights into the microstructure and mechanical properties evolution of hydration products in cementitious materials incorporating GGBFS. J. Sustain. Cen.-Based Mater..

[63] Xu C.W., Ni W., Li K.Q., Zhang S., Xu D. (2021). Activation mechanisms of three types of industrial by-product gypsums on steel slag–granulated blast furnace slag-based binders. Constr. Build. Mater..

[64] Pan Q.L. (2004). Discussion of hydration mechanism of granulated blastfurnace slag. Cement.

[65] Wu H., Ni W., Cui X.W., Wang S. (2014). Preparation of concrete sleeper using hot steaming steel slag with low autogenous shrinkage. Trans. Mater. Heat Treat..

[66] Zhang S.Q., Shi T.Y., Ni W., Li K., Gao W., Wang K., Zhang Y. (2021). The mechanism of hydrating and solidifying green mine fill materials using circulating fluidized bed fly ash-slag-based agent. J. Hazard. Mater..

[67] Chen W., Peng R., Straub C., Yuan B. (2020). Promoting the performance of one-part alkali-activated slag using fine lead-zinc mine tailings. Constr. Build. Mater..

[68] Otsuki N., Miyazato S., Yodsudjai W. (2003). Influence of Recycled Aggregate on Interfacial Transition Zone, Strength, Chloride Penetration and Carbonation of Concrete. J. Mater. Civ. Eng..

[69] Yang K., Zhong M.Q., Magee B., Yang C.H., Wang C., Zhu X.H., Zhang Z.L. (2017). Investigation of effects of Portland cement fineness and alkali content on concrete plastic shrinkage cracking. Constr. Build. Mater..

[70] Chappex T., Scrivener K. (2012). Alkali fixation of C–S–H in blended cement pastes and its relation to alkali silica reaction. Cem. Concr. Res..

